# New species need characters: comments on recently described apicomplexan parasites from Australia

**DOI:** 10.1186/s13071-019-3424-9

**Published:** 2019-04-16

**Authors:** D. James Harris

**Affiliations:** 1 Centro de Investigação em Biodiversidade e Recursos Genéticos (CIBIO), InBIO Laboratório Associado, Universidade do Porto, Campus Agrário de Vairão, 4485-661 Vairão, Portugal; 2 Departamento de Biologia, Faculdade de Ciências, Universidade do Porto, R. Campo Alegre s/n, 4169-007 Porto, Portugal

**Keywords:** *Babesia*, *Theileria*, *Hepatozoon*, Companion animals, Ticks

## Abstract

Screening of ticks from companion animals from Australia using genetic tools has recently been used to identify and name eight new species of Apicomplexan parasites. However, the diagnosis of these was based on genetic distinctiveness and phylogenetic relationships rather than determination of specific characters, which is required by the International Code of Zoological Nomenclature. The recently proposed names are therefore invalid. The use of informal names is necessary until formal valid descriptions are available.

## Letter to the Editor

The Apicomplexa are a large phylum of parasitic alveolates. Despite their enormous public health and economic importance, in terms of biodiversity, the Apicomplexa is considered the least-known group of all, with as little as 0.1% of species formally described [1]. Molecular tools should help overcome this “Linnean shortfall” but such studies have also lagged behind those of free-living organisms [2]. The screening of ticks from Australia, and the identification and description of eight new species of parasites [3] demonstrates both the value and the need for this kind of approach.

However, for any species name to be available, it must satisfy the provisions of the *International Code of Zoological Nomenclature* (hereafter the *Code*). In particular, “To be available, every new name published after 1930 … must be accompanied by a description or definition that states in words characters that are purported to differentiate the taxon” (Article 13.1.1). In all cases, Greay et al. [3] mention only genetic distinctiveness and position within a clade in the sections related to diagnosis. As such, the names lack a description as required by the *Code*, and the names proposed, i.e. *Babesia lohae*, *Babesia mackerrasorum*, *Hepatozoon banethi*, *Hepatozoon ewingi*, *Theileria apogeana*, *Theileria palmeri*, *Theileria paparinii* and *Theileria worthingtonorum* are *nomina nuda*, and thus unavailable.

It should be stressed that this is not just a technicality. Given the few molecular studies of many groups of apicomplexan parasites it is highly likely that further screening will recover additional haplotypes for the *18S* rRNA marker used. To which species would these be assigned? Haplotypes falling between two species would represent additional new species, or variants of one already proposed? Because genetic distances are a continuum, without defined characters linked to the proposed names there is no way to place new sequences unequivocally within the taxonomic framework. Similarly, phylogenetic relationships and clade composition would be expected to change with additional data, making it impossible to associate new data with the proposed names. While these are general issues regarding the need for characters to define new species, the situation would be particularly complex in this case, where only *18S* rRNA gene sequences were used to differentiate forms. This is a slow-evolving gene, and in some cases distinct species of the Apicomplexa have been shown to be identical for this marker, for example *Sarcocystis wobeseri*, *Sarcocystis calchasi* and *Sarcocystis halieti* [4]. Given that host specificity is also not high, with single individual vertebrate hosts having been reported with multiple infections (e.g. [5]), it is also possible that the current *18S* rRNA sequences and vertebrate host information are not specific for a single parasite species.

This is not to automatically suggest that the parasites identified by [3] do not represent valid taxa. Although candidate species can be left unnamed pending morphological assessments, this in itself may lead to long time lags before the description of new species after first identification, in turn prolonging the Linnean shortfall. Alternatively, molecular characters can be used to satisfy article 13.1.1 of the *Code*, so long as they are explicitly stated in words. In the meantime, the names proposed by [3] should not be used to avoid taxonomic instability, and these parasites should only be referred to informally until valid descriptions are available.
